# Factors Affecting Human Papillomavirus Vaccine Trends in the United States of America

**DOI:** 10.7759/cureus.42617

**Published:** 2023-07-28

**Authors:** Deepa Vasireddy, Thevasha Sathiyakumar, Sumona Mondal, Shantanu Sur

**Affiliations:** 1 Department of Pediatrics, Pediatric Group of Acadiana, Lafayette, USA; 2 Department of Mathematics, Clarkson University, Potsdam, USA; 3 Department of Biology, Clarkson University, Potsdam, USA

**Keywords:** human papillomavirus vaccination, gardasil, hpv epidemiology, immunization schedule, cancer cervical

## Abstract

Background

Routine immunization of both girls and boys starting from nine years of age with the human papillomavirus (HPV) vaccine is the current recommendation. The objective of this retrospective study using National Health and Nutrition Examination Survey data was to evaluate the influence of sociodemographic factors on the series initiation and completion of the HPV vaccine from 2011 to 2020.

Methodology

The chi-square test was used to examine the statistical significance of the association between categorical variables and receipt of the HPV vaccine. The Cochran-Armitage test for trend was employed to assess the statistical significance of temporal trends in risk factors associated with rates of HPV vaccination. These trends were further quantified by a significant rate ratio by comparing them against the most recent survey years.

Results

HPV vaccine uptake was higher in the 9-14-year age group across survey years and had increased for both males and females over that time. The first dose of the HPV vaccine was most likely to be received by the 11-18-year age group. In the most recent survey of 2017-2020, the highest number of vaccination series completion was achieved for Gardasil®.

Conclusions

Improved physician efforts and strategies to vaccinate males, low socioeconomic strata patients, and ethnic minorities in more numbers are needed.

## Introduction

Prevention

There are three human papillomavirus (HPV) vaccines. The two-valent HPV vaccine, Cervarix®, was licensed for use in females but is no longer available in the United States. The four-valent HPV vaccine, Gardasil®, was licensed for use in females and males and is also no longer available in the United States. The nine-valent HPV vaccine, Gardasil 9®, was approved in February 2015 for routine immunization of males and females and since the end of 2016 is the one currently used in the United States for vaccinating both sexes for the approved age groups [1,2]. According to the vaccine safety data link, a statistically significant risk for any of the prespecified adverse events following the vaccine has not been found [1].

As Gardasil 9® has been in use in the United States since late 2016, there has been reassuring safety monitoring data so far on the vaccine. The vaccine was studied in clinical trials with more than 15,000 males and females. It provides protection against 6, 11, 16, 18, 31, 33, 45, 52, and 58 types of HPV. HPV types 16, 18, 31, 33, 45, 52, and 58 cause vulvar, vaginal, cervical, penile, anal, and oropharyngeal cancers. Condyloma acuminata or genital warts are caused by HPV types 6 and 11. The vaccine also offers protection against the precancerous lesions of the vulvar, vaginal, cervical, and anal regions [3].

Screening

The 2018 United States Preventive Services Task Force guidelines recommend screening with cervical cytology alone every three years for women aged 21 to 29 years. Cervical cancer screening in those younger than 21 years is not recommended. With the undertaking of HPV vaccination, the HPV infections and precancer lesions in the 21-24-year age group were lowered considerably, which has led to the screening recommendation by the American Cancer Society to start at a later age of 25 years [4].

Human papillomavirus disease burden globally and in the United States

In 2020, cervical cancer was the seventh most common cancer contributing to new cancer cases and the ninth most common cancer contributing to cancer deaths globally. In the United States, the incidence and mortality rate of cervical cancer in 2020 were 28.3 and 2.1 per 100,000 people, respectively [5,6]. In 2021, according to the National Cancer Center Surveillance, Epidemiology, and End Results Program, there were an estimated 4,290 cervical cancer deaths in the United States, contributing to 0.7% of all cancer deaths [5].

Despite having effective vaccines, the cancer burden can be reduced more efficiently by improving vaccine uptake. We undertook this study to investigate the sociodemographic factors affecting vaccine uptake, which could enable the formulation of targeted measures that can be taken to further improve vaccination coverage among the population.

## Materials and methods

Study design and population

The data of this study were obtained from the National Health and Nutrition Examination Survey (NHANES) 2011-2020, a nationally representative survey of the resident, civilian, non-institutionalized US population that collects health examination data [7]. The NHANES is conducted annually, with data being made available every two years. In this study, we analyzed four consecutive NHANES cycles where the subject numbers who responded to the questionnaires were (excluding the subjects having missing variables) 5,680, 6,014, 5,780, and 8,851 in 2011-2012, 2013-2014, 2015-2016, 2017-2020, respectively.

Study measures

The objective of evaluating the influence of sociodemographic factors on the series initiation and completion of the HPV vaccine over time was investigated based on the responses to four main survey questions provided in Table 1.

**Table 1:** Survey questions about the HPV vaccine included in the household interview for female and male participants NHANES (2011-2020). HPV: human papillomavirus; NHANES: National Health and Nutrition Examination Survey

Survey questions	Responses
Have you ever received one or more doses of the HPV vaccine?	Yes/No/Refused/Don’t know
How old were you when you received your first dose of the HPV vaccine?	Range of values
How many doses of HPV vaccine have you ever received?	1 dose/2 doses/3 doses/Refused/Don’t know
Which of the HPV vaccines did you receive? (Survey question on vaccine type was given to female respondents)	Cervarix/Gardasil/Gardasil 9/Unknown Gardasil/Refused/Don’t know

This study mainly focuses on the impact of sociodemographic variables, namely, sex, age, ethnicity, education level, and poverty-income (PI) ratio, as provided in Table 2. Each categorical variable was stratified into different groups, as depicted in Table 2.

**Table 2:** Sociodemographic variables used in the study.

Study variable	Study categories used
Age	1. 9–14 years 2. 15 years and older and 1. 9–10 years 2. 11–18 years 3. 19 years
Sex	1. Female 2. Male
Ethnicity	1. MA: Mexican American 2. NHB: Non-Hispanic Black 3. NHW: Non-Hispanic White 4. OH: Other-Hispanic 5. OR: Other race
Education	1. College degree & above 2. High school graduate 3. Less than high school 4. Unknown
PI ratio	1. <1.46: Low income 2. 1.46–4.83: Moderate income 3. >4.83: High income

The PI ratio was categorized based on population distribution of PI ratio by quartiles as provided by Palar et al. [8], where PI ratio <1.46 (25th percentile) corresponds to low income, PI ratio between 1.46 (25th percentile) and 4.83 (75th percentile) corresponds to moderate income, and PI ratio >4.83 (75th percentile) corresponds to higher income.

The survey responses to question 1 (Table 1) were used to assess the trends in the uptake of the HPV vaccine over time. Survey question 2 (Table 1) was utilized to look into the likelihood of vaccination uptake across different age groups, categorized as 9-10 years, 11-18 years, and 19 years. As a result, the effects of each variable on the stratified age groups were examined, with the exception of education as it is associated with age groups. Furthermore, the information on HPV vaccine series completion from 2011 to 2020 and vaccine trends over time was obtained from questions 3 and 4, respectively (Table 1). This information about all three vaccine types, namely, Cervarix®, Gardasil®, and Gardasil 9®, among females was available only for the survey year 2017-2020. It is known that the number of required doses for Gardasil® and Gardasil 9® are 2 and 3 for the respective age groups of 9-14 years and 15 years. Furthermore, as the survey question on the type of HPV vaccine received was provided only for females in the NHANES survey, our study investigates the HPV vaccine series completion for females over time under age groups, ethnicity, and education.

Data analysis

The statistical significance of the association between sociodemographic categories and HPV vaccination was determined using the chi-square test [9,10]. The Cochran-Armitage test for trend [11] was conducted to assess the statistical significance of temporal trends in the association of sociodemographic categories with rates of HPV vaccination (based on survey question 1). Additionally, these trends were quantified by rate ratios, and statistical tests were conducted to compare the most recent survey years (2017-2020) to the other years (2015-2016, 2013-2014, and 2011-2012).

## Results

Four specific survey questions regarding HPV vaccination (Table 1) were utilized in this study to conduct our analysis. The findings obtained from investigating each question are summarized below.

Findings based on HPV vaccine uptake

Based on survey question 1, a higher percentage of subjects in the age group of 9-14 years received the HPV vaccine compared with the age group of ≥15 years in all survey years (Tables 3-6).

**Table 3:** The number (percentage) of responses provided for the survey year 2017-2020 NHANES survey question “Have you ever received one or more doses of the HPV vaccine?” and the relationship between categorical variables with HPV vaccine received (based on “Yes” and “No” responses) using the chi-square test. MA: Mexican American; NHB: Non-Hispanic Black; NHW: Non-Hispanic White; OH: Other Hispanic; OR: Other race; HPV: human papillomavirus; NHANES: National Health and Nutrition Examination Survey; PI: poverty-income

Variables	Categories	Responses on receiving HPV vaccination, n (%)				
		Yes, N (%)	No, N (%)	Chi-square test p-value based only on Yes/No respondents	Don’t know, N (%)	Refused, N (%)
Age (years)	9–14	505 (27.30)	1,180 (63.90)	<0.001	163 (8.80)	0 (0.00)
	≥15	1,249 (17.80)	5,135 (73.30)		617 (8.80)	2 (0.10)
Sex	Female	1,059 (23.20)	3,194 (69.90)	<0.001	313 (6.90)	1 (0.00)
	Male	695 (16.20)	3121 (72.90)		467 (10.90)	1 (0.00)
Ethnicity	MA	231 (18.20)	889 (70.10)	<0.001	149 (11.70)	0 (0.00)
	NHB	489 (21.00)	1,702 (72.90)		142 (6.10)	1 (0.00)
	NHW	551 (20.70)	1,933 (72.50)		182 (6.80)	1 (0.00)
	OH	173 (19.80)	600 (68.70)		100 (11.50)	0 (0.00)
	OR	310 (18.10)	1,191 (69.70)		207 (12.10)	0 (0.00)
Education	College degree and above	500 (14.30)	2,687 (77.10)	<0.001	298 (8.50)	2 (0.10)
	High school graduate	139 (10.20)	1,112 (81.60)		112 (8.20)	0 (0.00)
	Less than high school	64 (6.70)	815 (85.30)		76 (8.00)	0 (0.00)
	Unknown	1051 (34.50)	1,701 (55.80)		294 (9.70)	0 (0.00)
PI ratio	Low income	628 (22.5)	1,902 (68.00)	<0.001	267 (9.50)	0 (0.00)
	Moderate income	664 (19.4)	2,501 (73.00)		259 (7.60)	0 (0.00)
	High income	462 (17.6)	1,912 (72.70)		254 (9.70)	2 (0.10)

**Table 4:** The number (percentage) of responses provided for the survey year 2015-2016 NHANES survey question “Have you ever received one or more doses of the HPV vaccine?” and the relationship between categorical variables with HPV vaccine received (based on “Yes” and “No” responses) using the chi-square test. MA: Mexican American; NHB: Non-Hispanic Black; NHW: Non-Hispanic White; OH: Other Hispanic; OR: Other race; HPV: human papillomavirus; NHANES: National Health and Nutrition Examination Survey; PI: poverty-income

Variables	Categories		Responses on receiving the HPV vaccination, n (%)			
		Yes, N (%)	No, N (%)	Chi-square test p-value based only on Yes/No respondents	Don’t know, N (%)	Refused, N (%)
Age (years)	9–14	285 (24.00)	792 (66.70)	<0.001	10 (8.80)	5 (0.40)
	≥15	681 (14.80)	3,539(77.10)		37 (8.10)	2 (0.00)
Sex	Female	618 (20.60)	2,182 (72.80)	<0.001	193 (6.40)	3 (0.10)
	Male	348 (12.50)	2,149 (77.20)		283 (10.20)	4 (0.10)
Ethnicity	MA	187 (16.90)	817 (73.80)	<0.001	103 (9.30)	0 (0.00)
	NHB	238 (18.90)	908 (72.10)		113 (90)	1(0.10)
	NHW	287 (17.20)	1,289 (77.50)		82 (4.90)	6 (0.40)
	OH	120 (16.20)	554 (74.60)		69 (9.30)	0 (0.00)
	OR	134 (13.30)	763 (75.80)		109 (10.80)	0 (0.00)
Education	College degree and above	264 (11.90)	1,815 (81.60)	<0.001	144 (6.50)	0 (0.00)
	High school graduate	68 (8.30)	674 (82.60)		73 (8.90)	1 (0.10)
	Less than high school	21 (2.70)	692 (88.80)		65 (8.30)	1 (0.10)
	Unknown	613 (31.20)	1,150 (58.60)		194 (9.90)	5 (0.30)
PI ratio	Low income	355 (18.10)	1,410 (72.00)	<0.001	192 (9.80)	0 (0.00)
	Moderate income	402 (16.70)	1,822 (75.90)		177 (7.40)	1 (0.00)
	High income	209 (14.70)	1,099 (77.30)		107 (7.50)	6 (0.40)

**Table 5:** The number (percentage) of responses provided for the survey year 2013-2014 NHANES survey question “Have you ever received one or more doses of the HPV vaccine?” and the relationship between categorical variables with HPV vaccine received (based on “Yes” and “No” responses) using the chi-square test. MA: Mexican American; NHB: Non-Hispanic Black; NHW: Non-Hispanic White; OH: Other Hispanic; OR: Other race; HPV: human papillomavirus; NHANES: National Health and Nutrition Examination Survey; PI: poverty-income

Variables	Categories		Responses on receiving HPV vaccination, n (%)			
		Yes, N (%)	No, N (%)	Chi-square test p-value based only on Yes/No respondents	Don’t know, N (%)	Refused, N (%)
Age (years)	9–14	256 (21.00)	890 (73.10)	<0.001	71 (5.80)	0 (0.00)
	≥15	621 (12.90)	3,915 (81.60)		261 (5.40)	0 (0.00)
Sex	Female	600 (19.40)	2,363 (76.50)	<0.001	125 (4.00)	0 (0.00)
	Male	277 (9.50)	2,442 (83.50)		207 (7.10)	0 (0.00)
Ethnicity	MA	197 (18.80)	776 (74.10)	<0.001	74 (7.10)	0 (0.00)
	NHB	214 (16.30)	1,029 (78.50)		68 (5.20)	0 (0.00)
	NHW	256 (12.10)	1,753 (83.20)		98 (4.70)	0 (0.00)
	OH	85 (15.00)	441 (77.90)		40 (7.10)	0 (0.00)
	OR	125 (12.70)	806 (82.00)		52 (5.30)	0 (0.00)
Education	College degree and above	200 (8.70)	1,994 (86.50)	<0.001	112 (4.90)	0 (0.00)
	High school graduate	51 (5.90)	780 (90.40)		32 (3.70)	0 (0.00)
	Less than high school	41 (5.400)	671 (89.00)		42 (5.60)	0 (0.00)
	Unknown	585 (28)	1,360 (65.00)		146 (7.00)	0 (0.00)
PI ratio	Low income	403 (16.80)	1,848 (76.90)	<0.001	151 (6.30)	0 (0.00)
	Moderate income	306 (13.80)	1,799 (80.90)		118 (5.30)	0 (0.00)
	High income	168 (12.10)	1,158 (83.40)		63 (4.50)	0 (0.00)

**Table 6:** The number (percentage) of responses provided for the survey year 2011-2012 NHANES survey question “Have you ever received one or more doses of the HPV vaccine?” and the relationship between categorical variables with HPV vaccine received (based on “Yes” and “No” responses) using the chi-square test. MA: Mexican American; NHB: Non-Hispanic Black; NHW: Non-Hispanic White; OH: Other Hispanic; OR: Other race; HPV: human papillomavirus; NHANES: National Health and Nutrition Examination Survey; PI: poverty-income

Variables	Categories			Responses on receiving HPV vaccination, n (%)			
		Yes, N (%)	No, N (%)		Chi-square test p-value based only on Yes/No respondents	Don’t know, N (%)	Refused, N (%)
Age (years)	09-14	190 (16.50)	906 (78.70)		<0.001	55 (4.80)	0 (0.00)
	≥15	425 (9.40)	3,861 (85.30)			240 (5.30)	3 (0.10)
Sex	Female	482 (16.80)	2,269 (79.10)		<0.001	115 (4.00)	3 (0.10)
	Male	133 (4.70)	2,498 (88.90)			180 (6.40)	0 (0.00)
Ethnicity	MA	84 (11.10)	641 (84.70)		<0.001	32 (4.20)	0 (0.00)
	NHB	191 (12.40)	1,269 (82.30)			81 (5.30)	0 (0.00)
	NHW	164 (9.50)	1,503 (87.00)			60 (3.50)	0 (0.00)
	OH	74 (12.80)	462 (80.10)			41 (7.10)	0 (0.00)
	OR	102 (9.50)	892 (82.70)			81 (7.50)	3 (0.30)
Education	College degree and above	159 (7.10)	1,983 (88.30)		<0.001	105 (4.70)	0 (0.00)
	High school graduate	25 (3.20)	713 (92.20)			33 (4.30)	2 (0.30)
	Less than high school	21 (2.80)	693 (92.50)			35 (4.70)	0 (0.00)
	Unknown	410 (21.50)	1,378 (72.10)			122 (6.40)	1 (0.10)
PI ratio	Low income	299 (12.80)	1,903 (81.20)		<0.001	140 (6.00)	0 (0.00)
	Moderate income	191 (9.50)	1,738 (86.30)			86 (4.30)	3 (0.10)
	High income	72 (8.40)	761 (88.90)			23 (2.70)	0 (0.00)

In addition, the vaccination rate had almost doubled from 2011-2012 (9.4%) to 2017-2020 (17.8%) for the age group of 15 years, while for the age group of 9-14 years, the observed increase was approximately 1.66 times (Table 7).

**Table 7:** The Cochran-Armitage test to detect temporal linear trends in HPV vaccination rate with the sociodemographic categories. The rate ratio represents the comparison of the survey years (2011-2012, 2013-2014, 2015-2016) against the most recent survey year (2017-2020, reference year). ***: p < 0.01; **: p < 0.05; *: p < 0.10. MA: Mexican American, NHB: Non-Hispanic Black, NHW: Non-Hispanic White, OH: Other Hispanic, OR: Other race; HPV: human papillomavirus; PI: poverty-income

Variables	P-value	2011–2012	2013–2014	2015–2016
Age				
9–14	<0.001	1.66***	1.30***	1.05
≥15	<0.001	1.90***	1.38***	1.29***
Sex				
Female	<0.001	1.38***	1.19***	1.12**
Male	<0.001	3.43***	1.71***	1.30***
Ethnicity				
MA	<0.001	1.64***	0.97	1.08
NHB	<0.001	1.69***	1.28***	1.11
NHW	<0.001	2.18***	1.70***	1.20**
OH	<0.001	1.55***	1.32**	1.23*
OR	<0.001	1.92***	1.43***	1.36***
Education				
College degree and above	<0.001	2.03***	1.65***	1.21**
High school graduate	<0.001	3.15***	1.73***	1.22
Less than high school	0.001	2.39***	1.23	2.49***
Unknown	<0.001	1.61***	1.23***	1.10*
PI ratio				
Low income	<0.001	1.76***	1.34***	1.24***
Moderate income	<0.001	2.05***	1.41***	1.16**
High income	<0.001	2.09***	1.45***	1.29***

The percentage of the HPV vaccine received by females had increased from 16.8% in 2011-2012 to 23.2% in 2017-2020 (Tables 3, 6), indicating 1.38 fold increase(Table 7). A corresponding change in the vaccination rate among males was more remarkable, which increased from 4.7% to 16.2% (Tables 3, 6), implying that males in the survey year 2017-2020 were almost thrice more likely to have been vaccinated than in 2011-2012 (Table 7).

Among the ethnic groups, Non-Hispanic Black (NHB) received the highest percentage of the HPV vaccine compared to other ethnic groups in survey years 2017-2020 and 2015-2016 (Tables 3, 4), while Mexican American (MA) and Other-Hispanic (OH) had the highest vaccination rates in survey years 2013-2014 and 2011-2012, respectively (Tables 5, 6). The percentage of vaccine recipients in the Non-Hispanic White (NHW) and Other race (OR) categories almost doubled from 9.5% in 2011-2012 to 20.70% and 18.10%, respectively, in 2017-2020 (Tables 3, 6, 7). Other ethnic groups also showed an increase in the vaccination rate during the same period.

Comparing vaccination rates against education, having a “college degree and above” consistently showed higher vaccination rates for all survey years over the “high school graduate” and “less than high school” categories. The improvement in vaccination rate over the survey years was also observed in all categories, with the highest improvement (3.15 times) being found in the high school graduate categories. It should also be noted that the category with “unknown” education information showed the highest vaccination rate in the survey data. The percentage of the HPV vaccine received by the unknown education category had increased from 21.5% in 2011-2012 to 34.5% in 2017-2020 (Tables 3, 6), indicating a 1.61 times increase (Table 7). The percentage of the HPV vaccine received by the unknown education category had increased from 28% in 2013-2014 to 34.5% in 2017-2020 (Tables 3, 5), indicating a 1.23 times increase (Table 7). The percentage of the HPV vaccine received by the unknown education category had increased from 31.2% in 2015-2016 to 34.5% in 2017-2020 (Tables 3, 4), indicating a 1.10 times increase (Table 7).

Under the PI ratio category, the percentage of HPV vaccine received in the low-income category was the highest compared to other PI ratio categories for the complete period 2011-2020 (Tables 3-6). It was noted that both categories of moderate income and high income in 2017-2020 were twice more likely to have been vaccinated than in 2011-2012 (Table 7).

**Figure 1:**
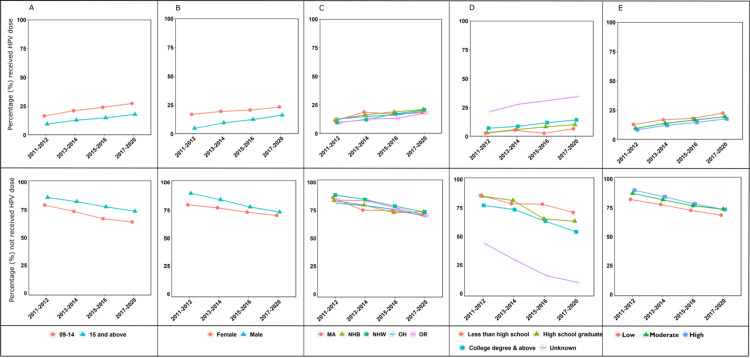
Plots to visualize temporal trends of (A) gender, (B) ethnicity, (C) age group, (D) education, and (E) poverty-income ratio over four survey years for individuals who responded received at least one dose of the HPV vaccine and not received any HPV vaccine doses. HPV: human papillomavirus

Furthermore, based on the Cochran-Armitage test result (p < 0.001, as in Table 7) and Figure 1, the significant upward temporal trend was evident irrespective of categories in each variable.

Findings based on the age of the first dose of the HPV vaccine series

**Figure 2:**
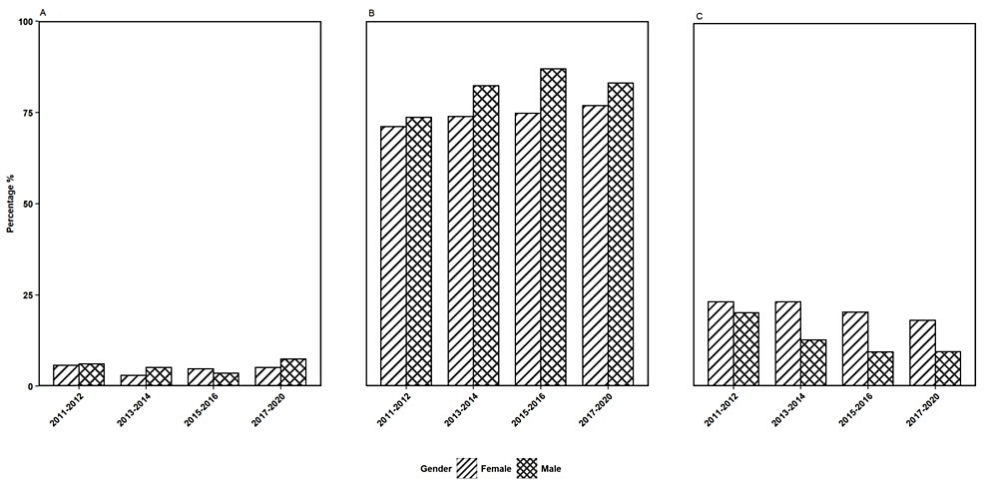
The distribution of gender of the participants who received the first dose of the HPV vaccine in three age groups of (A) 9-10 years, (B) 11-18 years, (C) 19 years or older across four survey years considered in this study. HPV: human papillomavirus

**Figure 3:**
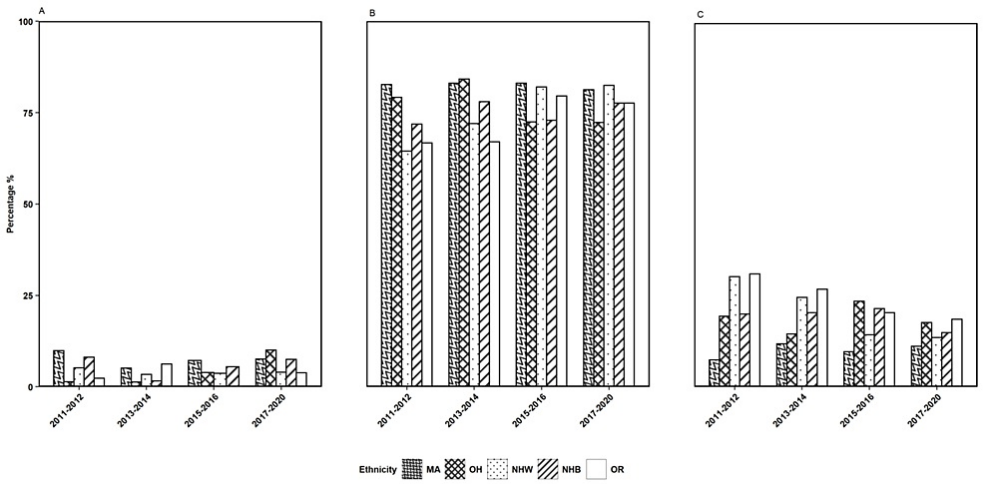
The distributions of ethnicity of the participants who received the first dose of the HPV vaccine in three age groups of (A) 9-10 years, (B) 11-18 years, and (C) 19 years and older across four survey years considered in the study. HPV: human papillomavirus

**Figure 4:**
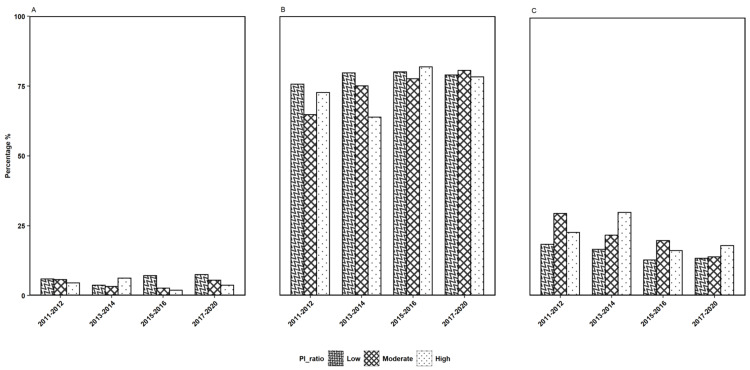
The distributions of the poverty-income ratio of the participants who received the first dose of the HPV vaccine in three age groups of (A) 9-10 years, (B) 11-18 years, and (C) 19 years and older across four survey years considered in this study. HPV: human papillomavirus

As the survey data indicates an association of HPV vaccination rate with age groups, we established an age group stratification for gender, ethnicity, and PI ratio to further evaluate the impact of each variable using the responses to survey question 2. The observations depicted in Figures 2-4 show the gender, ethnicity, and PI ratio distributions of the respondents who received the first dose of the HPV vaccine for the three age groups, respectively.

The prominent observation is that irrespective of the differences in gender, ethnicity, and PI ratio the first dosage of the HPV vaccine was most likely to be received by the age group of 11-18 years, followed by 19 years, and 9-10 years age groups. This observation corroborates the fact that the HPV vaccine was recommended by the Advisory Committee on Immunization Practices (ACIP) at the age of 11-12 years and vaccination through the age of 26 years if not adequately vaccinated when younger.

Further, in the particular age group of 11-18 years, the percentage of females who had taken the first dose of the HPV vaccine was noted to be highest in the survey year 2017-2020, and for males, the percentage was noted to be highest in 2015-2016 (Figure 2). Considering ethnic groups, the percentage of the first dosage of the HPV vaccine received in 11-18 years for ethnic groups MA, NHB, and OH was the highest in the survey year 2013-2014, whereas, for the ethnic groups OR and NHW, it was the highest in the respective years 2015-2016 and 2017-2020 (Figure 3). Under income-based categories, the low-income category had a higher percentage of the first dose of vaccination in the 11-18 years age group for the survey years 2013-2014 and 2015-2016, whereas, for the middle-income category and the high-income category, it was the highest in the respective years 2017-2020 and 2015-2016 (Figure 4).

Findings based on the HPV vaccine series completion

Based on survey questions 3 and 4, Table 8 and Table 9 summarize the information on HPV vaccine series completion for all three vaccine types (Cervarix®, Gardasil®, and Gardasil 9®) in females.

**Table 8:** The criteria for HPV vaccine dose completion for each age group based on the type of HPV vaccine. HPV: human papillomavirus

Vaccine type	Age group (in years)	Number of doses
Cervarix®	All age groups	3
Gardasil®, Gardasil 9®	9–14	2
Gardasil®, Gardasil 9®	≥15	3

**Table 9:** The number of females who completed vaccine requirements specific to vaccine type for each variable in 2017-2020. MA: Mexican American, NHB: Non-Hispanic Black, NHW: Non-Hispanic White, OH: Other Hispanic, OR: Other race; PI: poverty-income

Completion of doses based on variables	Categories	Cervarix® Series completion (N)	Gardasil® Series completion (N)	Gardasil 9® Series completion (N)
Age (in years)	9–14	3	33	12
	≥15	17	163	7
Ethnicity	MA	0	21	3
	NHB	8	40	3
	NHW	7	93	8
	OH	2	15	0
	OR	3	27	5
PI ratio	Low income	9	50	5
	Moderate income	5	78	5
	High income	6	48	4

It was noted that in the most recent survey of 2017-2020, the highest number of vaccination series completion was achieved for Gardasil®. In the same survey, it was also found that the series completion of Gardasil® and Cervarix® were considerably higher in the age group of ≥15 years compared with 9-14 years but vice versa for the Gardasil 9® vaccine. Under the ethnicity, the highest number of vaccine series completion of Cervarix® was seen in NHB, whereas NHW showed the highest number of vaccine series completion of Gardasil®, Gardasil 9®. Under the income-based categories, the moderate-income group showed the highest number of series completion of Gardasil®.

Additionally, Table 10 showed that for three survey years between 2011 and 2016, the higher number of series completion of both Gardasil® and Cervarix® was evident for the age group ≥15 years.

**Table 10:** The number of females who completed vaccine requirements specific to vaccine type (Cervarix, Gardasil) for each variable in 2015-2016, 2013-2014, and 2011-2012. MA: Mexican American; NHB: Non-Hispanic Black; NHW: Non-Hispanic White; OH: Other Hispanic; OR: Other race; PI: poverty-income

Completion of doses based on variables	Categories	2015–2016		2013–2014		2011–2012	
		Cervarix® Series completion (N)	Gardasil® Series completion (N)	Cervarix® Series completion (N)	Gardasil® Series completion (N)	Cervarix® Series completion (N)	Gardasil® Series completion (N)
Age (in years)	9–14	7	42	1	51	2	55
	≥15	18	180	12	197	7	132
Ethnicity	MA	8	24	5	36	2	12
	NHB	7	47	5	63	2	51
	NHW	5	95	2	92	0	85
	OH	4	24	0	23	3	14
	OR	1	32	1	34	2	25
PI ratio	Low income	8	56	7	86	4	62
	Moderate income	12	97	4	86	4	56
	High income	5	40	2	47	1	34

Based on ethnicity, NHW showed higher number series completion of Gardasil® for all three survey years between 2011-2016, whereas the higher number series completion of Cervarix® based on ethnic groups varied over the survey years. In regard to the PI ratio, the moderate income group showed a higher number of series completion of Gardasil® in 2015-2016 with a gradual increase over the survey years. According to the series completion of Cervarix®, the highest number was noted in the low-income group and moderate-income group for the respective survey years 2013-2014 and 2015-2016.

## Discussion

There are certain myths and misinformation around the HPV vaccine that lots of families harbor which seem to hinder vaccination efforts for HPV. One concern is that the child may start having sex early on in life but studies have shown this is not the case [12]. Some families are not aware that boys require the vaccine. It has been shown to prevent genital warts, penile, anal, and oropharyngeal cancers in men [13]. The HPV vaccine causing infertility is another concern raised by parents and confirmed by studies to contribute to vaccine hesitancy. HPV vaccine has not been shown to have any effect on fertility. However, precancerous or cancerous lesions caused by HPV and the treatment for them could affect the women’s ability to have children [3]. One study found that healthcare providers themselves are less comfortable vaccinating the younger populations compared to older adolescents. Professional organizations’ endorsement of the vaccine also plays a pivotal role [14]. In a survey-based study, secondary acceptance of the HPV vaccine seemed to be influenced by the quality of recommendation at the initial discussion, satisfaction with communication with the provider, more knowledge about the vaccine acquired over time, and the child getting older [15]. The ACIP recommended routine use of the four-valent HPV vaccine (HPV4; Gardasil®, Merck & Co. Inc.) in males aged 11 or 12 years on October 25, 2011 [16]. One cross-sectional study based on The National Immunization Survey-Teen (NIS-Teen), conducted by the Centers for Disease Control and Prevention in 2011, noted that 59% of parents of adolescent girls reported that they had been recommended the HPV vaccine for their child by the provider compared to only 14% parents of the boys [17]. We do not have data on the reason for refusal of the vaccine which would have been essential in determining where to focus reform efforts.

According to the NIS-Teen in 2017, 51% of adolescents had not completed their HPV vaccine schedule, and adolescents in rural areas were lagging behind their urban area counterparts in vaccination rates. In 2020, between ages 13 and 17 years, only 61.4% of females and 56% of males were fully vaccinated against HPV. For this age group, Rhode Island was the leading state for the highest coverage rate for fully vaccinated males and females and Mississippi had the lowest coverage rate nationwide [18]. In our study, when considering the series completion for females, it was evident that in all survey years, the highest number of series completion was achieved for Gardasil®, and the series completion of Gardasil was considerably higher in the age group of ≥15 years. The HPV vaccine has high immunogenicity. A month after completing the vaccination series, 98% of the recipients develop an antibody response to the HPV types in the vaccine [19]. In June 2020, it was determined that 55% of the WHO Member States had introduced HPV vaccination, with the Americas and Europe leading. Globally, the vaccine introduction has been unequal. Some populous countries have not yet introduced the vaccine leading to a lower global vaccination rate among males and females.

According to one study in 2019, it was found that globally, 15% of girls and 4% of boys were fully vaccinated and 20% and 5% received at least one dose of the vaccine respectively [20]. In another study, it was observed that between 2008 and 2018, HPV vaccine awareness had declined over the years, where racial minorities, rural populations, males, those at and over 65 years of age, and those with the lowest educational and socioeconomic status had the lowest awareness [21]. In a study that collected data from 2008 to 2012 from the NIS-Teen, an upward trend of the initiation and completion of the HPV vaccine in girls aged less than 13 years of age was observed [22]. 

Our study also reveals that when considering the initiation of HPV, the percentage of receiving the first dose of the HPV vaccine was noted to be the highest in the age group 11-18 years. In the particular age group of 11-18 years, the percentage of females who had taken the first dose of the HPV vaccine was noted to be the highest in the survey year 2017-2020, and for males, the percentage was noted to be the highest in 2015-2016.

According to a systematic review in 2007, it was found that African American, Hispanic, and white ethnic groups had equal acceptance of the HPV vaccine [23]. Another study based on adolescent girls noted that completion of the vaccination was less likely in Blacks and Hispanics [24]. According to a study in 2013, girls and boys had HPV vaccine initiation rates of 57.3% and 34.6%, respectively, and completion rates of less than 40% and 15%, respectively [25]. Our study signifies an age group stratification for ethnicity in receiving the first dose of HPV. Considering ethnic groups, the percentage of first dosage of HPV vaccine received in 11-18 years for ethnic groups MA, NHB, and OH was the highest in the survey year 2013-2014, whereas, for the ethnic groups OR and NHW, it was highest in the respective years 2015-2016 and 2017-2020.

Our study results are in consensus with previous literature in regard to findings on age, gender, and ethnicity. In addition, the results of our study revealed hidden insights about the association and temporal trends of the risk factors education level and PI ratio with the receival of HPV vaccine. Missing data from survey subjects can contribute to non-respondent bias. Our study does show we have made an improvement in HPV vaccination coverage over the past 10 years but we are yet to reach our goal. As per Healthy People 2030, the current target is to immunize 80% of the adolescent population (13-15 years) with the HPV vaccine and 54.4% has been achieved as of 2020 [26].

The authors do recognize that the results obtained by a retrospective study are limited because they cannot prove a causal relationship and a true causal-effect link can only be established by properly conducted prospective studies where there is an option of taking care of biases of different kinds. However, a retrospective study can be helpful for gathering preliminary information and directing the design of upcoming prospective investigations.

## Conclusions

Various sociodemographic factors influence the series initiation and completion of the HPV vaccine. Physicians focus a lot on the diagnosis and treatment of cervical cancer but better education and more open conversations about the HPV vaccine as a preventative measure by the medical community is a much-needed step. Parental attitudes, beliefs, and barriers play a crucial role in vaccinating children and adolescents. Education at every well visit about the benefits starting even before the recommended age groups will help prime the families’ receptiveness to the vaccine at the appropriate time. Based on the findings of our study, males, low socioeconomic strata patients, populations with less than high school education, 9-10-year age group, and ethnic minorities are the groups that should be focused upon.
